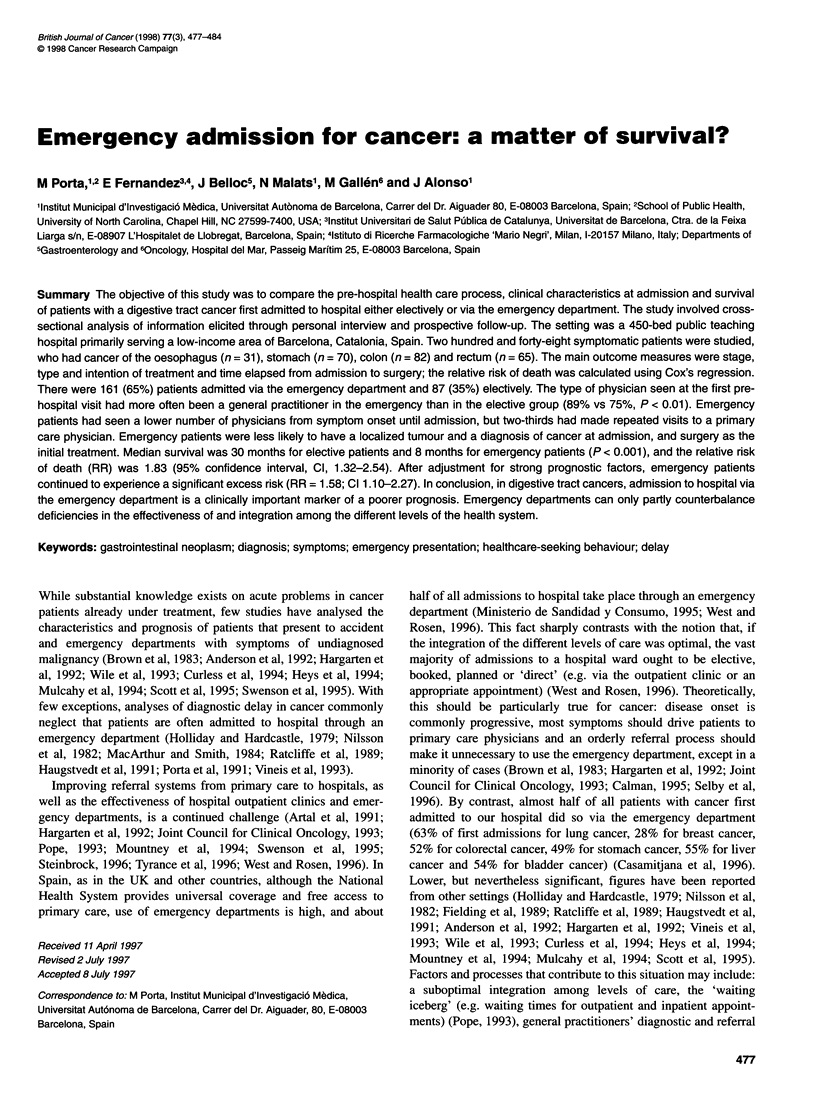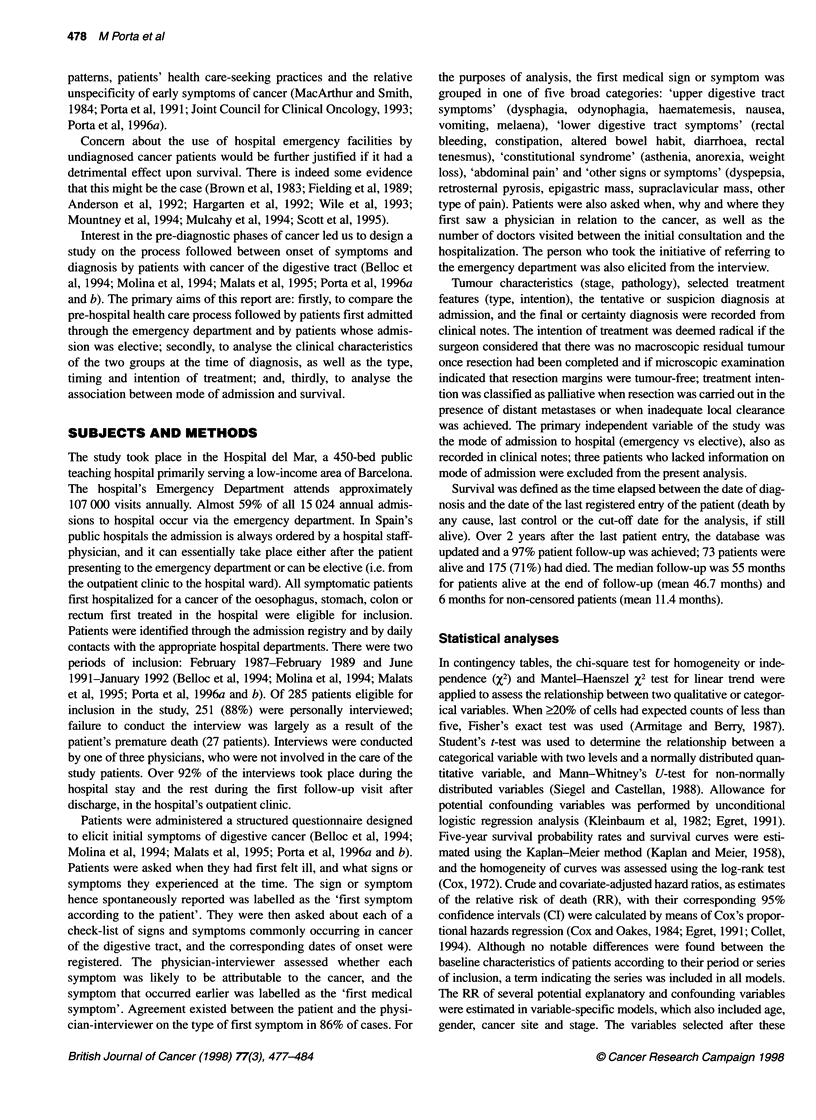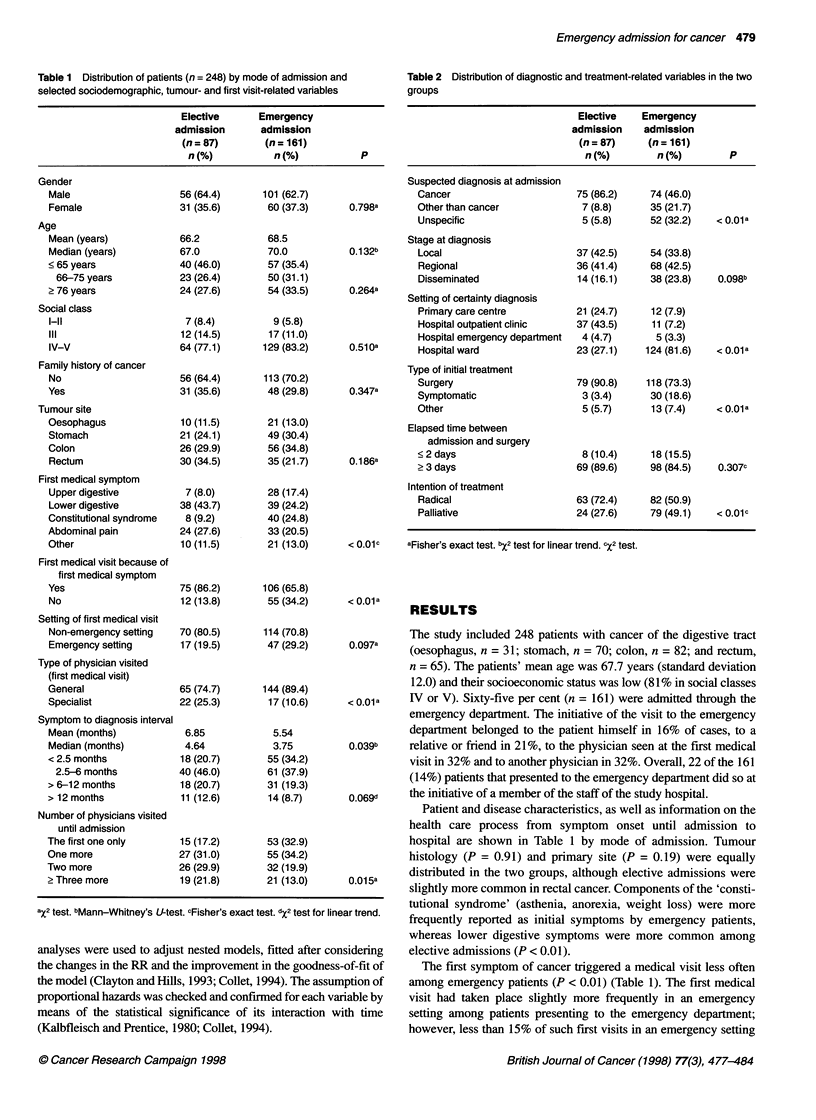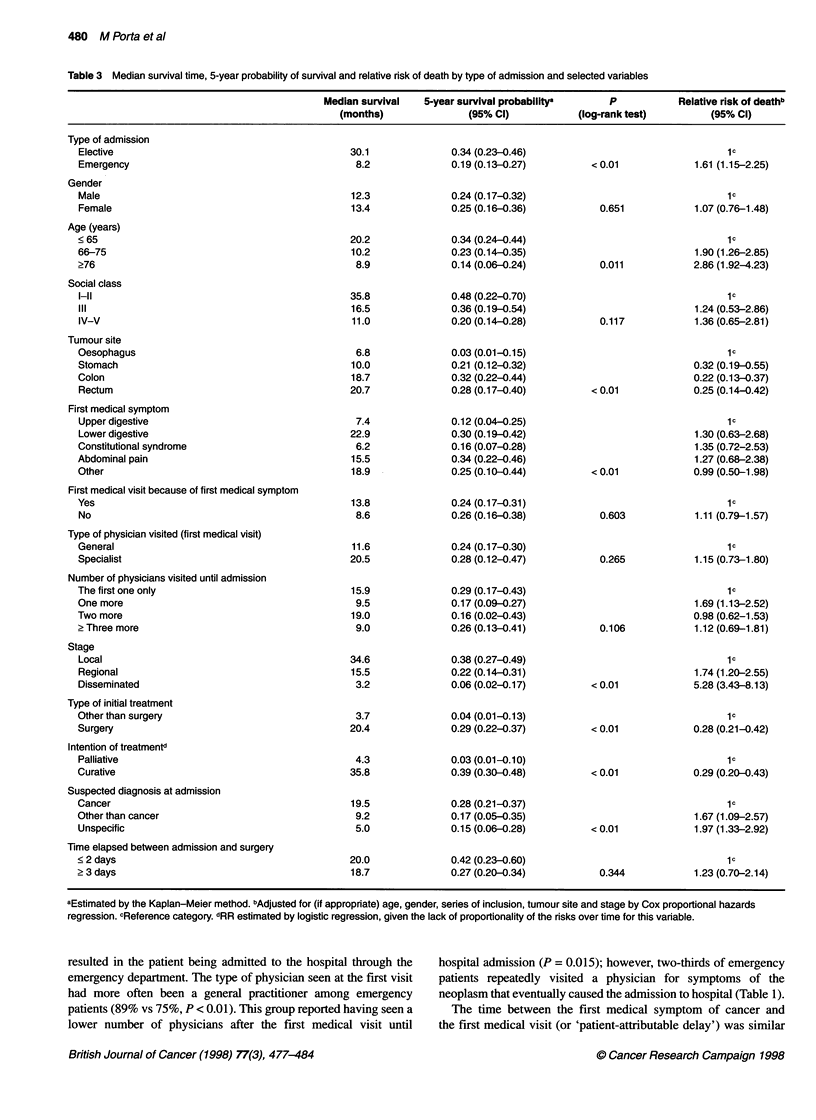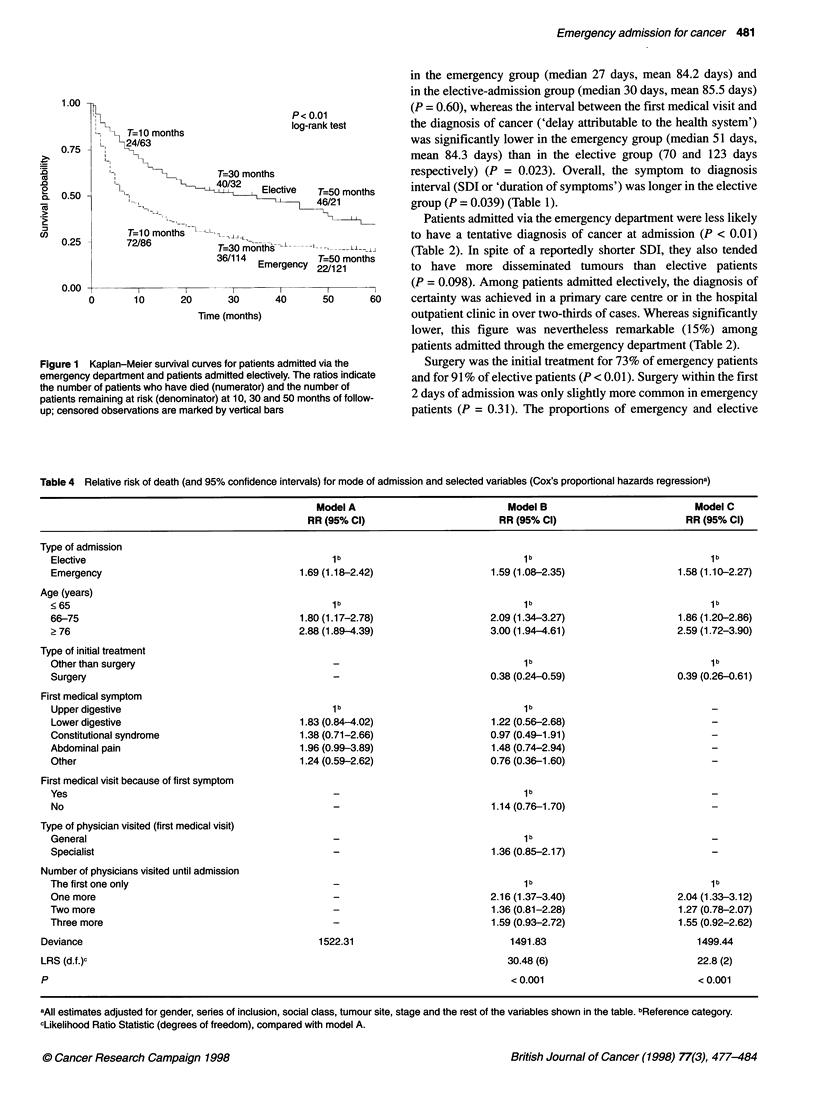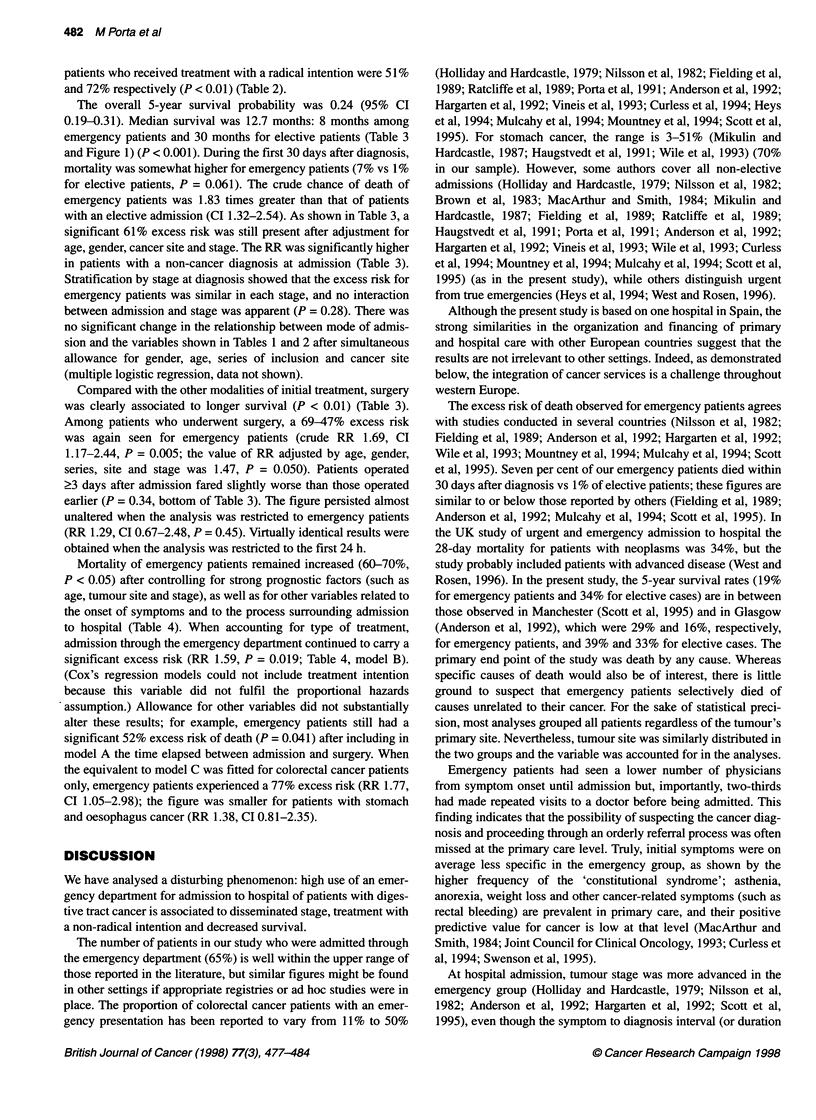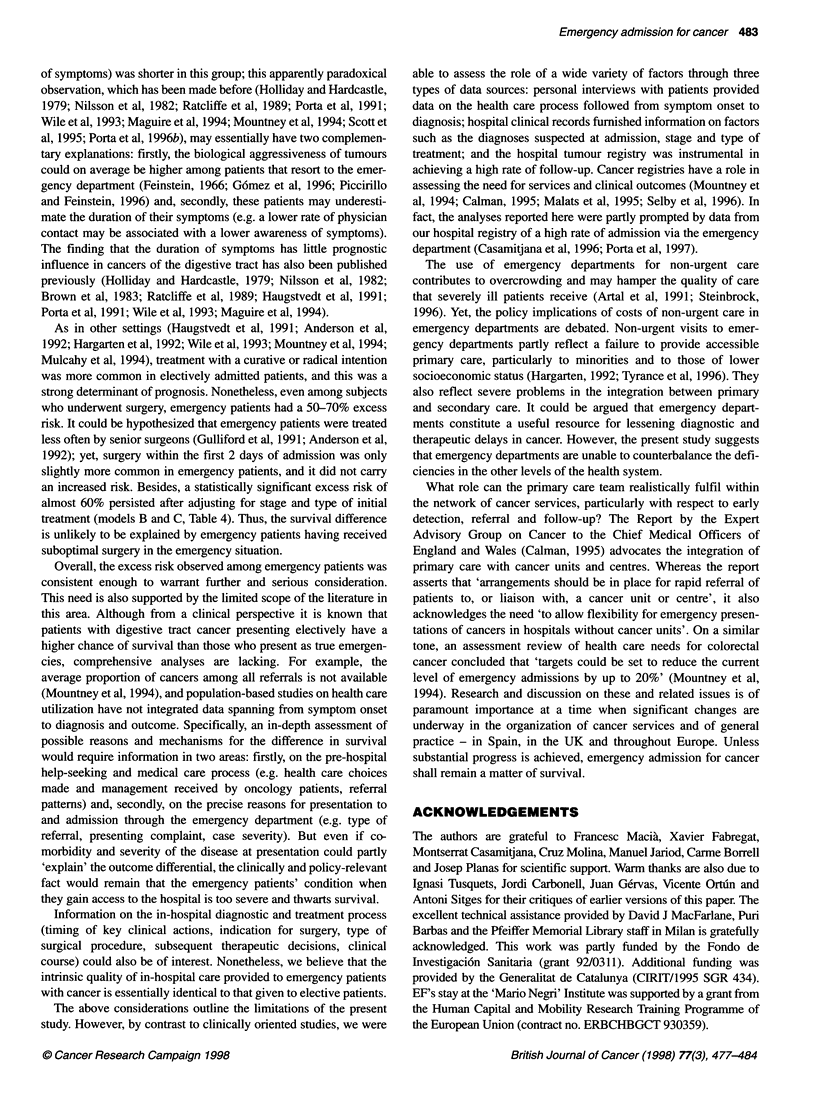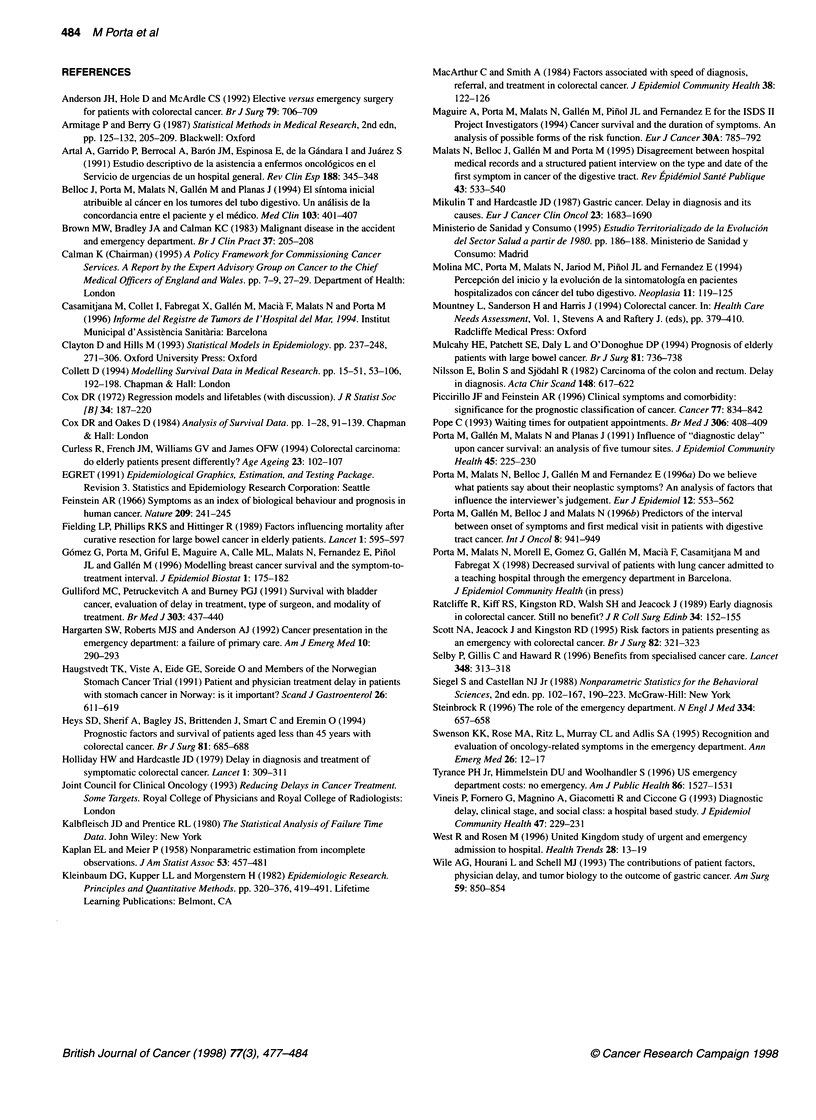# Emergency admission for cancer: a matter of survival?

**DOI:** 10.1038/bjc.1998.76

**Published:** 1998

**Authors:** M. Porta, E. Fernandez, J. Belloc, N. Malats, M. GallÃ©n, J. Alonso

**Affiliations:** Institut Municipal d'InvestigaciÃ³ MÃ¨dica, Universitat AutÃ²noma de Barcelona, Spain.

## Abstract

The objective of this study was to compare the pre-hospital health care process, clinical characteristics at admission and survival of patients with a digestive tract cancer first admitted to hospital either electively or via the emergency department. The study involved cross-sectional analysis of information elicited through personal interview and prospective follow-up. The setting was a 450-bed public teaching hospital primarily serving a low-income area of Barcelona, Catalonia, Spain. Two hundred and forty-eight symptomatic patients were studied, who had cancer of the oesophagus (n = 31), stomach (n = 70), colon (n = 82) and rectum (n = 65). The main outcome measures were stage, type and intention of treatment and time elapsed from admission to surgery; the relative risk of death was calculated using Cox's regression. There were 161 (65%) patients admitted via the emergency department and 87 (35%) electively. The type of physician seen at the first pre-hospital visit had more often been a general practitioner in the emergency than in the elective group (89% vs 75%, P < 0.01). Emergency patients had seen a lower number of physicians from symptom onset until admission, but two-thirds had made repeated visits to a primary care physician. Emergency patients were less likely to have a localized tumour and a diagnosis of cancer at admission, and surgery as the initial treatment. Median survival was 30 months for elective patients and 8 months for emergency patients (P < 0.001), and the relative risk of death (RR) was 1.83 (95% confidence interval, CI, 1.32-2.54). After adjustment for strong prognostic factors, emergency patients continued to experience a significant excess risk (RR = 1.58; CI 1.10-2.27). In conclusion, in digestive tract cancers, admission to hospital via the emergency department is a clinically important marker of a poorer prognosis. Emergency departments can only partly counterbalance deficiencies in the effectiveness of and integration among the different levels of the health system.


British Journal of Cancer (1998) 77(3), 477–484
© 1998 Cancer Research Campaign

### Emergency admission for cancer: a matter of survival?

**M Porta,[1,2] E Fernandez[3,4], J Belloc[5], N Malats[1], M Gallén[6] and J Alonso[1]**

[1]Institut Municipal d'Investigació Mèdica, Universitat Autònoma de Barcelona, Carrer del Dr. Aiguader 80, E-08003 Barcelona, Spain; [2]School of Public Health, University of North Carolina, Chapel Hill, NC 27599-7400, USA; [3]Institut Universitari de Salut Pública de Catalunya, Universitat de Barcelona, Ctra. de la Feixa Liarga s/n, E-08907 L'Hospitalet de Llobregat, Barcelona, Spain; [4]Istituto di Ricerche Farmacologiche 'Mario Negri', Milan, I-20157 Milano, Italy; Departments of [5]Gastroenterology and [6]Oncology, Hospital del Mar, Passeig Marítim 25, E-08003 Barcelona, Spain

**Summary** The objective of this study was to compare the pre-hospital health care process, clinical characteristics at admission and survival of patients with a digestive tract cancer first admitted to hospital either electively or via the emergency department. The study involved cross-sectional analysis of information elicited through personal interview and prospective follow-up. The setting was a 450-bed public teaching hospital primarily serving a low-income area of Barcelona, Catalonia, Spain. Two hundred and forty-eight symptomatic patients were studied, who had cancer of the oesophagus ($n = 31$), stomach ($n = 70$), colon ($n = 82$) and rectum ($n = 65$). The main outcome measures were stage, type and intention of treatment and time elapsed from admission to surgery; the relative risk of death was calculated using Cox's regression. There were 161 (65%) patients admitted via the emergency department and 87 (35%) electively. The type of physician seen at the first pre-hospital visit had more often been a general practitioner in the emergency than in the elective group (89% vs 75%, $P < 0.01$). Emergency patients had seen a lower number of physicians from symptom onset until admission, but two-thirds had made repeated visits to a primary care physician. Emergency patients were less likely to have a localized tumour and a diagnosis of cancer at admission, and surgery as the initial treatment. Median survival was 30 months for elective patients and 8 months for emergency patients ($P < 0.001$), and the relative risk of death (RR) was 1.83 (95% confidence interval, CI, 1.32–2.54). After adjustment for strong prognostic factors, emergency patients continued to experience a significant excess risk (RR = 1.58; CI 1.10–2.27). In conclusion, in digestive tract cancers, admission to hospital via the emergency department is a clinically important marker of a poorer prognosis. Emergency departments can only partly counterbalance deficiencies in the effectiveness of and integration among the different levels of the health system.

**Keywords:** gastrointestinal neoplasm; diagnosis; symptoms; emergency presentation; healthcare-seeking behaviour; delay

While substantial knowledge exists on acute problems in cancer patients already under treatment, few studies have analysed the characteristics and prognosis of patients that present to accident and emergency departments with symptoms of undiagnosed malignancy (Brown et al, 1983; Anderson et al, 1992; Hargarten et al, 1992; Wile et al, 1993; Curless et al, 1994; Heys et al, 1994; Mulcahy et al, 1994; Scott et al, 1995; Swenson et al, 1995). With few exceptions, analyses of diagnostic delay in cancer commonly neglect that patients are often admitted to hospital through an emergency department (Holliday and Hardcastle, 1979; Nilsson et al, 1982; MacArthur and Smith, 1984; Ratcliffe et al, 1989; Haugstvedt et al, 1991; Porta et al, 1991; Vineis et al, 1993).

Improving referral systems from primary care to hospitals, as well as the effectiveness of hospital outpatient clinics and emergency departments, is a continued challenge (Artal et al, 1991; Hargarten et al, 1992; Joint Council for Clinical Oncology, 1993; Pope, 1993; Mountney et al, 1994; Swenson et al, 1995; Steinbrock, 1996; Tyrance et al, 1996; West and Rosen, 1996). In Spain, as in the UK and other countries, although the National Health System provides universal coverage and free access to primary care, use of emergency departments is high, and about half of all admissions to hospital take place through an emergency department (Ministerio de Sandidad y Consumo, 1995; West and Rosen, 1996). This fact sharply contrasts with the notion that, if the integration of the different levels of care was optimal, the vast majority of admissions to a hospital ward ought to be elective, booked, planned or 'direct' (e.g. via the outpatient clinic or an appropriate appointment) (West and Rosen, 1996). Theoretically, this should be particularly true for cancer: disease onset is commonly progressive, most symptoms should drive patients to primary care physicians and an orderly referral process should make it unnecessary to use the emergency department, except in a minority of cases (Brown et al, 1983; Hargarten et al, 1992; Joint Council for Clinical Oncology, 1993; Calman, 1995; Selby et al, 1996). By contrast, almost half of all patients with cancer first admitted to our hospital did so via the emergency department (63% of first admissions for lung cancer, 28% for breast cancer, 52% for colorectal cancer, 49% for stomach cancer, 55% for liver cancer and 54% for bladder cancer) (Casamitjana et al, 1996). Lower, but nevertheless significant, figures have been reported from other settings (Holliday and Hardcastle, 1979; Nilsson et al, 1982; Fielding et al, 1989; Ratcliffe et al, 1989; Haugstvedt et al, 1991; Anderson et al, 1992; Hargarten et al, 1992; Vineis et al, 1993; Wile et al, 1993; Curless et al, 1994; Heys et al, 1994; Mountney et al, 1994; Mulcahy et al, 1994; Scott et al, 1995). Factors and processes that contribute to this situation may include: a suboptimal integration among levels of care, the 'waiting iceberg' (e.g. waiting times for outpatient and inpatient appointments) (Pope, 1993), general practitioners' diagnostic and referral

*Received 11 April 1997*
*Revised 2 July 1997*
*Accepted 8 July 1997*

*Correspondence to:* M Porta, Institut Municipal d'Investigació Mèdica, Universitat Autónoma de Barcelona, Carrer del Dr. Aiguader, 80, E-08003 Barcelona, Spain patterns, patients' health care-seeking practices and the relative unspecificity of early symptoms of cancer (MacArthur and Smith, 1984; Porta et al, 1991; Joint Council for Clinical Oncology, 1993; Porta et al, 1996*a*).

Concern about the use of hospital emergency facilities by undiagnosed cancer patients would be further justified if it had a detrimental effect upon survival. There is indeed some evidence that this might be the case (Brown et al, 1983; Fielding et al, 1989; Anderson et al, 1992; Hargarten et al, 1992; Wile et al, 1993; Mountney et al, 1994; Mulcahy et al, 1994; Scott et al, 1995).

Interest in the pre-diagnostic phases of cancer led us to design a study on the process followed between onset of symptoms and diagnosis by patients with cancer of the digestive tract (Belloc et al, 1994; Molina et al, 1994; Malats et al, 1995; Porta et al, 1996*a* and *b*). The primary aims of this report are: firstly, to compare the pre-hospital health care process followed by patients first admitted through the emergency department and by patients whose admission was elective; secondly, to analyse the clinical characteristics of the two groups at the time of diagnosis, as well as the type, timing and intention of treatment; and, thirdly, to analyse the association between mode of admission and survival.

### SUBJECTS AND METHODS

The study took place in the Hospital del Mar, a 450-bed public teaching hospital primarily serving a low-income area of Barcelona. The hospital's Emergency Department attends approximately 107 000 visits annually. Almost 59% of all 15 024 annual admissions to hospital occur via the emergency department. In Spain's public hospitals the admission is always ordered by a hospital staff-physician, and it can essentially take place either after the patient presenting to the emergency department or can be elective (i.e. from the outpatient clinic to the hospital ward). All symptomatic patients first hospitalized for a cancer of the oesophagus, stomach, colon or rectum first treated in the hospital were eligible for inclusion. Patients were identified through the admission registry and by daily contacts with the appropriate hospital departments. There were two periods of inclusion: February 1987–February 1989 and June 1991–January 1992 (Belloc et al, 1994; Molina et al, 1994; Malats et al, 1995; Porta et al, 1996*a* and *b*). Of 285 patients eligible for inclusion in the study, 251 (88%) were personally interviewed; failure to conduct the interview was largely as a result of the patient's premature death (27 patients). Interviews were conducted by one of three physicians, who were not involved in the care of the study patients. Over 92% of the interviews took place during the hospital stay and the rest during the first follow-up visit after discharge, in the hospital's outpatient clinic.

Patients were administered a structured questionnaire designed to elicit initial symptoms of digestive cancer (Belloc et al, 1994; Molina et al, 1994; Malats et al, 1995; Porta et al, 1996*a* and *b*). Patients were asked when they had first felt ill, and what signs or symptoms they experienced at the time. The sign or symptom hence spontaneously reported was labelled as the 'first symptom according to the patient'. They were then asked about each of a check-list of signs and symptoms commonly occurring in cancer of the digestive tract, and the corresponding dates of onset were registered. The physician-interviewer assessed whether each symptom was likely to be attributable to the cancer, and the symptom that occurred earlier was labelled as the 'first medical symptom'. Agreement existed between the patient and the physician-interviewer on the type of first symptom in 86% of cases. For the purposes of analysis, the first medical sign or symptom was grouped in one of five broad categories: 'upper digestive tract symptoms' (dysphagia, odynophagia, haematemesis, nausea, vomiting, melaena), 'lower digestive tract symptoms' (rectal bleeding, constipation, altered bowel habit, diarrhoea, rectal tenesmus), 'constitutional syndrome' (asthenia, anorexia, weight loss), 'abdominal pain' and 'other signs or symptoms' (dyspepsia, retrosternal pyrosis, epigastric mass, supraclavicular mass, other type of pain). Patients were also asked when, why and where they first saw a physician in relation to the cancer, as well as the number of doctors visited between the initial consultation and the hospitalization. The person who took the initiative of referring to the emergency department was also elicited from the interview.

Tumour characteristics (stage, pathology), selected treatment features (type, intention), the tentative or suspicion diagnosis at admission, and the final or certainty diagnosis were recorded from clinical notes. The intention of treatment was deemed radical if the surgeon considered that there was no macroscopic residual tumour once resection had been completed and if microscopic examination indicated that resection margins were tumour-free; treatment intention was classified as palliative when resection was carried out in the presence of distant metastases or when inadequate local clearance was achieved. The primary independent variable of the study was the mode of admission to hospital (emergency vs elective), also as recorded in clinical notes; three patients who lacked information on mode of admission were excluded from the present analysis.

Survival was defined as the time elapsed between the date of diagnosis and the date of the last registered entry of the patient (death by any cause, last control or the cut-off date for the analysis, if still alive). Over 2 years after the last patient entry, the database was updated and a 97% patient follow-up was achieved; 73 patients were alive and 175 (71%) had died. The median follow-up was 55 months for patients alive at the end of follow-up (mean 46.7 months) and 6 months for non-censored patients (mean 11.4 months).

### Statistical analyses

In contingency tables, the chi-square test for homogeneity or independence ($\chi^2$) and Mantel–Haenszel $\chi^2$ test for linear trend were applied to assess the relationship between two qualitative or categorical variables. When ≥20% of cells had expected counts of less than five, Fisher's exact test was used (Armitage and Berry, 1987). Student's *t*-test was used to determine the relationship between a categorical variable with two levels and a normally distributed quantitative variable, and Mann–Whitney's *U*-test for non-normally distributed variables (Siegel and Castellan, 1988). Allowance for potential confounding variables was performed by unconditional logistic regression analysis (Kleinbaum et al, 1982; Egret, 1991). Five-year survival probability rates and survival curves were estimated using the Kaplan–Meier method (Kaplan and Meier, 1958), and the homogeneity of curves was assessed using the log-rank test (Cox, 1972). Crude and covariate-adjusted hazard ratios, as estimates of the relative risk of death (RR), with their corresponding 95% confidence intervals (CI) were calculated by means of Cox's proportional hazards regression (Cox and Oakes, 1984; Egret, 1991; Collet, 1994). Although no notable differences were found between the baseline characteristics of patients according to their period or series of inclusion, a term indicating the series was included in all models. The RR of several potential explanatory and confounding variables were estimated in variable-specific models, which also included age, gender, cancer site and stage. The variables selected after these

© Cancer Research Campaign 1998

**Table 1** Distribution of patients ($n$ = 248) by mode of admission and selected sociodemographic, tumour- and first visit-related variables

| | Elective admission ($n$ = 87) $n$ (%) | Emergency admission ($n$ = 161) $n$ (%) | $P$ |
|---|---|---|---|
| Gender | | | |
| Male | 56 (64.4) | 101 (62.7) | |
| Female | 31 (35.6) | 60 (37.3) | 0.798[a] |
| Age | | | |
| Mean (years) | 66.2 | 68.5 | |
| Median (years) | 67.0 | 70.0 | 0.132[b] |
| ≤ 65 years | 40 (46.0) | 57 (35.4) | |
| 66–75 years | 23 (26.4) | 50 (31.1) | |
| ≥ 76 years | 24 (27.6) | 54 (33.5) | 0.264[a] |
| Social class | | | |
| I–II | 7 (8.4) | 9 (5.8) | |
| III | 12 (14.5) | 17 (11.0) | |
| IV–V | 64 (77.1) | 129 (83.2) | 0.510[a] |
| Family history of cancer | | | |
| No | 56 (64.4) | 113 (70.2) | |
| Yes | 31 (35.6) | 48 (29.8) | 0.347[a] |
| Tumour site | | | |
| Oesophagus | 10 (11.5) | 21 (13.0) | |
| Stomach | 21 (24.1) | 49 (30.4) | |
| Colon | 26 (29.9) | 56 (34.8) | |
| Rectum | 30 (34.5) | 35 (21.7) | 0.186[a] |
| First medical symptom | | | |
| Upper digestive | 7 (8.0) | 28 (17.4) | |
| Lower digestive | 38 (43.7) | 39 (24.2) | |
| Constitutional syndrome | 8 (9.2) | 40 (24.8) | |
| Abdominal pain | 24 (27.6) | 33 (20.5) | |
| Other | 10 (11.5) | 21 (13.0) | < 0.01[c] |
| First medical visit because of first medical symptom | | | |
| Yes | 75 (86.2) | 106 (65.8) | |
| No | 12 (13.8) | 55 (34.2) | < 0.01[a] |
| Setting of first medical visit | | | |
| Non-emergency setting | 70 (80.5) | 114 (70.8) | |
| Emergency setting | 17 (19.5) | 47 (29.2) | 0.097[a] |
| Type of physician visited (first medical visit) | | | |
| General | 65 (74.7) | 144 (89.4) | |
| Specialist | 22 (25.3) | 17 (10.6) | < 0.01[a] |
| Symptom to diagnosis interval | | | |
| Mean (months) | 6.85 | 5.54 | |
| Median (months) | 4.64 | 3.75 | 0.039[b] |
| < 2.5 months | 18 (20.7) | 55 (34.2) | |
| 2.5–6 months | 40 (46.0) | 61 (37.9) | |
| > 6–12 months | 18 (20.7) | 31 (19.3) | |
| > 12 months | 11 (12.6) | 14 (8.7) | 0.069[d] |
| Number of physicians visited until admission | | | |
| The first one only | 15 (17.2) | 53 (32.9) | |
| One more | 27 (31.0) | 55 (34.2) | |
| Two more | 26 (29.9) | 32 (19.9) | |
| ≥ Three more | 19 (21.8) | 21 (13.0) | 0.015[a] |

[a]$\chi^2$ test. [b]Mann–Whitney's *U*-test. [c]Fisher's exact test. [d]$\chi^2$ test for linear trend.

analyses were used to adjust nested models, fitted after considering the changes in the RR and the improvement in the goodness-of-fit of the model (Clayton and Hills, 1993; Collet, 1994). The assumption of proportional hazards was checked and confirmed for each variable by means of the statistical significance of its interaction with time (Kalbfleisch and Prentice, 1980; Collet, 1994).

**Table 2** Distribution of diagnostic and treatment-related variables in the two groups

| | Elective admission ($n$ = 87) $n$ (%) | Emergency admission ($n$ = 161) $n$ (%) | $P$ |
|---|---|---|---|
| Suspected diagnosis at admission | | | |
| Cancer | 75 (86.2) | 74 (46.0) | |
| Other than cancer | 7 (8.8) | 35 (21.7) | |
| Unspecific | 5 (5.8) | 52 (32.2) | < 0.01[a] |
| Stage at diagnosis | | | |
| Local | 37 (42.5) | 54 (33.8) | |
| Regional | 36 (41.4) | 68 (42.5) | |
| Disseminated | 14 (16.1) | 38 (23.8) | 0.098[b] |
| Setting of certainty diagnosis | | | |
| Primary care centre | 21 (24.7) | 12 (7.9) | |
| Hospital outpatient clinic | 37 (43.5) | 11 (7.2) | |
| Hospital emergency department | 4 (4.7) | 5 (3.3) | |
| Hospital ward | 23 (27.1) | 124 (81.6) | < 0.01[a] |
| Type of initial treatment | | | |
| Surgery | 79 (90.8) | 118 (73.3) | |
| Symptomatic | 3 (3.4) | 30 (18.6) | |
| Other | 5 (5.7) | 13 (7.4) | < 0.01[a] |
| Elapsed time between admission and surgery | | | |
| ≤ 2 days | 8 (10.4) | 18 (15.5) | |
| ≥ 3 days | 69 (89.6) | 98 (84.5) | 0.307[c] |
| Intention of treatment | | | |
| Radical | 63 (72.4) | 82 (50.9) | |
| Palliative | 24 (27.6) | 79 (49.1) | < 0.01[c] |

[a]Fisher's exact test. [b]$\chi^2$ test for linear trend. [c]$\chi^2$ test.

### RESULTS

The study included 248 patients with cancer of the digestive tract (oesophagus, $n$ = 31; stomach, $n$ = 70; colon, $n$ = 82; and rectum, $n$ = 65). The patients' mean age was 67.7 years (standard deviation 12.0) and their socioeconomic status was low (81% in social classes IV or V). Sixty-five per cent ($n$ = 161) were admitted through the emergency department. The initiative of the visit to the emergency department belonged to the patient himself in 16% of cases, to a relative or friend in 21%, to the physician seen at the first medical visit in 32% and to another physician in 32%. Overall, 22 of the 161 (14%) patients that presented to the emergency department did so at the initiative of a member of the staff of the study hospital.

Patient and disease characteristics, as well as information on the health care process from symptom onset until admission to hospital are shown in Table 1 by mode of admission. Tumour histology ($P$ = 0.91) and primary site ($P$ = 0.19) were equally distributed in the two groups, although elective admissions were slightly more common in rectal cancer. Components of the 'constitutional syndrome' (asthenia, anorexia, weight loss) were more frequently reported as initial symptoms by emergency patients, whereas lower digestive symptoms were more common among elective admissions ($P$ < 0.01).

The first symptom of cancer triggered a medical visit less often among emergency patients ($P$ < 0.01) (Table 1). The first medical visit had taken place slightly more frequently in an emergency setting among patients presenting to the emergency department; however, less than 15% of such first visits in an emergency setting

© Cancer Research Campaign 1998 

Table 3 Median survival time, 5-year probability of survival and relative risk of death by type of admission and selected variables

| | Median survival (months) | 5-year survival probability[a] (95% CI) | P (log-rank test) | Relative risk of death[b] (95% CI) |
|---|---|---|---|---|
| Type of admission | | | | |
| Elective | 30.1 | 0.34 (0.23–0.46) | | 1[c] |
| Emergency | 8.2 | 0.19 (0.13–0.27) | < 0.01 | 1.61 (1.15–2.25) |
| Gender | | | | |
| Male | 12.3 | 0.24 (0.17–0.32) | | 1[c] |
| Female | 13.4 | 0.25 (0.16–0.36) | 0.651 | 1.07 (0.76–1.48) |
| Age (years) | | | | |
| ≤ 65 | 20.2 | 0.34 (0.24–0.44) | | 1[c] |
| 66–75 | 10.2 | 0.23 (0.14–0.35) | | 1.90 (1.26–2.85) |
| ≥76 | 8.9 | 0.14 (0.06–0.24) | 0.011 | 2.86 (1.92–4.23) |
| Social class | | | | |
| I–II | 35.8 | 0.48 (0.22–0.70) | | 1[c] |
| III | 16.5 | 0.36 (0.19–0.54) | | 1.24 (0.53–2.86) |
| IV–V | 11.0 | 0.20 (0.14–0.28) | 0.117 | 1.36 (0.65–2.81) |
| Tumour site | | | | |
| Oesophagus | 6.8 | 0.03 (0.01–0.15) | | 1[c] |
| Stomach | 10.0 | 0.21 (0.12–0.32) | | 0.32 (0.19–0.55) |
| Colon | 18.7 | 0.32 (0.22–0.44) | | 0.22 (0.13–0.37) |
| Rectum | 20.7 | 0.28 (0.17–0.40) | < 0.01 | 0.25 (0.14–0.42) |
| First medical symptom | | | | |
| Upper digestive | 7.4 | 0.12 (0.04–0.25) | | 1[c] |
| Lower digestive | 22.9 | 0.30 (0.19–0.42) | | 1.30 (0.63–2.68) |
| Constitutional syndrome | 6.2 | 0.16 (0.07–0.28) | | 1.35 (0.72–2.53) |
| Abdominal pain | 15.5 | 0.34 (0.22–0.46) | | 1.27 (0.68–2.38) |
| Other | 18.9 | 0.25 (0.10–0.44) | < 0.01 | 0.99 (0.50–1.98) |
| First medical visit because of first medical symptom | | | | |
| Yes | 13.8 | 0.24 (0.17–0.31) | | 1[c] |
| No | 8.6 | 0.26 (0.16–0.38) | 0.603 | 1.11 (0.79–1.57) |
| Type of physician visited (first medical visit) | | | | |
| General | 11.6 | 0.24 (0.17–0.30) | | 1[c] |
| Specialist | 20.5 | 0.28 (0.12–0.47) | 0.265 | 1.15 (0.73–1.80) |
| Number of physicians visited until admission | | | | |
| The first one only | 15.9 | 0.29 (0.17–0.43) | | 1[c] |
| One more | 9.5 | 0.17 (0.09–0.27) | | 1.69 (1.13–2.52) |
| Two more | 19.0 | 0.16 (0.02–0.43) | | 0.98 (0.62–1.53) |
| ≥ Three more | 9.0 | 0.26 (0.13–0.41) | 0.106 | 1.12 (0.69–1.81) |
| Stage | | | | |
| Local | 34.6 | 0.38 (0.27–0.49) | | 1[c] |
| Regional | 15.5 | 0.22 (0.14–0.31) | | 1.74 (1.20–2.55) |
| Disseminated | 3.2 | 0.06 (0.02–0.17) | < 0.01 | 5.28 (3.43–8.13) |
| Type of initial treatment | | | | |
| Other than surgery | 3.7 | 0.04 (0.01–0.13) | | 1[c] |
| Surgery | 20.4 | 0.29 (0.22–0.37) | < 0.01 | 0.28 (0.21–0.42) |
| Intention of treatment[d] | | | | |
| Palliative | 4.3 | 0.03 (0.01–0.10) | | 1[c] |
| Curative | 35.8 | 0.39 (0.30–0.48) | < 0.01 | 0.29 (0.20–0.43) |
| Suspected diagnosis at admission | | | | |
| Cancer | 19.5 | 0.28 (0.21–0.37) | | 1[c] |
| Other than cancer | 9.2 | 0.17 (0.05–0.35) | | 1.67 (1.09–2.57) |
| Unspecific | 5.0 | 0.15 (0.06–0.28) | < 0.01 | 1.97 (1.33–2.92) |
| Time elapsed between admission and surgery | | | | |
| ≤ 2 days | 20.0 | 0.42 (0.23–0.60) | | 1[c] |
| ≥ 3 days | 18.7 | 0.27 (0.20–0.34) | 0.344 | 1.23 (0.70–2.14) |

[a]Estimated by the Kaplan–Meier method. [b]Adjusted for (if appropriate) age, gender, series of inclusion, tumour site and stage by Cox proportional hazards regression. [c]Reference category. [d]RR estimated by logistic regression, given the lack of proportionality of the risks over time for this variable.

resulted in the patient being admitted to the hospital through the emergency department. The type of physician seen at the first visit had more often been a general practitioner among emergency patients (89% vs 75%, $P < 0.01$). This group reported having seen a lower number of physicians after the first medical visit until hospital admission ($P = 0.015$); however, two-thirds of emergency patients repeatedly visited a physician for symptoms of the neoplasm that eventually caused the admission to hospital (Table 1).

The time between the first medical symptom of cancer and the first medical visit (or 'patient-attributable delay') was similar

 © Cancer Research Campaign 1998

**Figure 1** Kaplan–Meier survival curves for patients admitted via the emergency department and patients admitted electively. The ratios indicate the number of patients who have died (numerator) and the number of patients remaining at risk (denominator) at 10, 30 and 50 months of follow-up; censored observations are marked by vertical bars in the emergency group (median 27 days, mean 84.2 days) and in the elective-admission group (median 30 days, mean 85.5 days) ($P = 0.60$), whereas the interval between the first medical visit and the diagnosis of cancer ('delay attributable to the health system') was significantly lower in the emergency group (median 51 days, mean 84.3 days) than in the elective group (70 and 123 days respectively) ($P = 0.023$). Overall, the symptom to diagnosis interval (SDI or 'duration of symptoms') was longer in the elective group ($P = 0.039$) (Table 1).

Patients admitted via the emergency department were less likely to have a tentative diagnosis of cancer at admission ($P < 0.01$) (Table 2). In spite of a reportedly shorter SDI, they also tended to have more disseminated tumours than elective patients ($P = 0.098$). Among patients admitted electively, the diagnosis of certainty was achieved in a primary care centre or in the hospital outpatient clinic in over two-thirds of cases. Whereas significantly lower, this figure was nevertheless remarkable (15%) among patients admitted through the emergency department (Table 2).

Surgery was the initial treatment for 73% of emergency patients and for 91% of elective patients ($P < 0.01$). Surgery within the first 2 days of admission was only slightly more common in emergency patients ($P = 0.31$). The proportions of emergency and elective

**Table 4** Relative risk of death (and 95% confidence intervals) for mode of admission and selected variables (Cox's proportional hazards regression[a])

| | Model A RR (95% CI) | Model B RR (95% CI) | Model C RR (95% CI) |
|---|---|---|---|
| Type of admission | | | |
| Elective | 1[b] | 1[b] | 1[b] |
| Emergency | 1.69 (1.18–2.42) | 1.59 (1.08–2.35) | 1.58 (1.10–2.27) |
| Age (years) | | | |
| ≤ 65 | 1[b] | 1[b] | 1[b] |
| 66–75 | 1.80 (1.17–2.78) | 2.09 (1.34–3.27) | 1.86 (1.20–2.86) |
| ≥ 76 | 2.88 (1.89–4.39) | 3.00 (1.94–4.61) | 2.59 (1.72–3.90) |
| Type of initial treatment | | | |
| Other than surgery | – | 1[b] | 1[b] |
| Surgery | – | 0.38 (0.24–0.59) | 0.39 (0.26–0.61) |
| First medical symptom | | | |
| Upper digestive | 1[b] | 1[b] | – |
| Lower digestive | 1.83 (0.84–4.02) | 1.22 (0.56–2.68) | – |
| Constitutional syndrome | 1.38 (0.71–2.66) | 0.97 (0.49–1.91) | – |
| Abdominal pain | 1.96 (0.99–3.89) | 1.48 (0.74–2.94) | – |
| Other | 1.24 (0.59–2.62) | 0.76 (0.36–1.60) | – |
| First medical visit because of first symptom | | | |
| Yes | – | 1[b] | – |
| No | – | 1.14 (0.76–1.70) | – |
| Type of physician visited (first medical visit) | | | |
| General | – | 1[b] | – |
| Specialist | – | 1.36 (0.85–2.17) | – |
| Number of physicians visited until admission | | | |
| The first one only | – | 1[b] | 1[b] |
| One more | – | 2.16 (1.37–3.40) | 2.04 (1.33–3.12) |
| Two more | – | 1.36 (0.81–2.28) | 1.27 (0.78–2.07) |
| Three more | – | 1.59 (0.93–2.72) | 1.55 (0.92–2.62) |
| Deviance | 1522.31 | 1491.83 | 1499.44 |
| LRS (d.f.)[c] | | 30.48 (6) | 22.8 (2) |
| *P* | | < 0.001 | < 0.001 |

[a]All estimates adjusted for gender, series of inclusion, social class, tumour site, stage and the rest of the variables shown in the table. [b]Reference category. [c]Likelihood Ratio Statistic (degrees of freedom), compared with model A.

patients who received treatment with a radical intention were 51% and 72% respectively ($P < 0.01$) (Table 2).

The overall 5-year survival probability was 0.24 (95% CI 0.19–0.31). Median survival was 12.7 months: 8 months among emergency patients and 30 months for elective patients (Table 3 and Figure 1) ($P < 0.001$). During the first 30 days after diagnosis, mortality was somewhat higher for emergency patients (7% vs 1% for elective patients, $P = 0.061$). The crude chance of death of emergency patients was 1.83 times greater than that of patients with an elective admission (CI 1.32–2.54). As shown in Table 3, a significant 61% excess risk was still present after adjustment for age, gender, cancer site and stage. The RR was significantly higher in patients with a non-cancer diagnosis at admission (Table 3). Stratification by stage at diagnosis showed that the excess risk for emergency patients was similar in each stage, and no interaction between admission and stage was apparent ($P = 0.28$). There was no significant change in the relationship between mode of admission and the variables shown in Tables 1 and 2 after simultaneous allowance for gender, age, series of inclusion and cancer site (multiple logistic regression, data not shown).

Compared with the other modalities of initial treatment, surgery was clearly associated to longer survival ($P < 0.01$) (Table 3). Among patients who underwent surgery, a 69–47% excess risk was again seen for emergency patients (crude RR 1.69, CI 1.17–2.44, $P = 0.005$; the value of RR adjusted by age, gender, series, site and stage was 1.47, $P = 0.050$). Patients operated ≥3 days after admission fared slightly worse than those operated earlier ($P = 0.34$, bottom of Table 3). The figure persisted almost unaltered when the analysis was restricted to emergency patients (RR 1.29, CI 0.67–2.48, $P = 0.45$). Virtually identical results were obtained when the analysis was restricted to the first 24 h.

Mortality of emergency patients remained increased (60–70%, $P < 0.05$) after controlling for strong prognostic factors (such as age, tumour site and stage), as well as for other variables related to the onset of symptoms and to the process surrounding admission to hospital (Table 4). When accounting for type of treatment, admission through the emergency department continued to carry a significant excess risk (RR 1.59, $P = 0.019$; Table 4, model B). (Cox's regression models could not include treatment intention because this variable did not fulfil the proportional hazards assumption.) Allowance for other variables did not substantially alter these results; for example, emergency patients still had a significant 52% excess risk of death ($P = 0.041$) after including in model A the time elapsed between admission and surgery. When the equivalent to model C was fitted for colorectal cancer patients only, emergency patients experienced a 77% excess risk (RR 1.77, CI 1.05–2.98); the figure was smaller for patients with stomach and oesophagus cancer (RR 1.38, CI 0.81–2.35).

### DISCUSSION

We have analysed a disturbing phenomenon: high use of an emergency department for admission to hospital of patients with digestive tract cancer is associated to disseminated stage, treatment with a non-radical intention and decreased survival.

The number of patients in our study who were admitted through the emergency department (65%) is well within the upper range of those reported in the literature, but similar figures might be found in other settings if appropriate registries or ad hoc studies were in place. The proportion of colorectal cancer patients with an emergency presentation has been reported to vary from 11% to 50% (Holliday and Hardcastle, 1979; Nilsson et al, 1982; Fielding et al, 1989; Ratcliffe et al, 1989; Porta et al, 1991; Anderson et al, 1992; Hargarten et al, 1992; Vineis et al, 1993; Curless et al, 1994; Heys et al, 1994; Mulcahy et al, 1994; Mountney et al, 1994; Scott et al, 1995). For stomach cancer, the range is 3–51% (Mikulin and Hardcastle, 1987; Haugstvedt et al, 1991; Wile et al, 1993) (70% in our sample). However, some authors cover all non-elective admissions (Holliday and Hardcastle, 1979; Nilsson et al, 1982; Brown et al, 1983; MacArthur and Smith, 1984; Mikulin and Hardcastle, 1987; Fielding et al, 1989; Ratcliffe et al, 1989; Haugstvedt et al, 1991; Porta et al, 1991; Anderson et al, 1992; Hargarten et al, 1992; Vineis et al, 1993; Wile et al, 1993; Curless et al, 1994; Mountney et al, 1994; Mulcahy et al, 1994; Scott et al, 1995) (as in the present study), while others distinguish urgent from true emergencies (Heys et al, 1994; West and Rosen, 1996).

Although the present study is based on one hospital in Spain, the strong similarities in the organization and financing of primary and hospital care with other European countries suggest that the results are not irrelevant to other settings. Indeed, as demonstrated below, the integration of cancer services is a challenge throughout western Europe.

The excess risk of death observed for emergency patients agrees with studies conducted in several countries (Nilsson et al, 1982; Fielding et al, 1989; Anderson et al, 1992; Hargarten et al, 1992; Wile et al, 1993; Mountney et al, 1994; Mulcahy et al, 1994; Scott et al, 1995). Seven per cent of our emergency patients died within 30 days after diagnosis vs 1% of elective patients; these figures are similar to or below those reported by others (Fielding et al, 1989; Anderson et al, 1992; Mulcahy et al, 1994; Scott et al, 1995). In the UK study of urgent and emergency admission to hospital the 28-day mortality for patients with neoplasms was 34%, but the study probably included patients with advanced disease (West and Rosen, 1996). In the present study, the 5-year survival rates (19% for emergency patients and 34% for elective cases) are in between those observed in Manchester (Scott et al, 1995) and in Glasgow (Anderson et al, 1992), which were 29% and 16%, respectively, for emergency patients, and 39% and 33% for elective cases. The primary end point of the study was death by any cause. Whereas specific causes of death would also be of interest, there is little ground to suspect that emergency patients selectively died of causes unrelated to their cancer. For the sake of statistical precision, most analyses grouped all patients regardless of the tumour's primary site. Nevertheless, tumour site was similarly distributed in the two groups and the variable was accounted for in the analyses.

Emergency patients had seen a lower number of physicians from symptom onset until admission but, importantly, two-thirds had made repeated visits to a doctor before being admitted. This finding indicates that the possibility of suspecting the cancer diagnosis and proceeding through an orderly referral process was often missed at the primary care level. Truly, initial symptoms were on average less specific in the emergency group, as shown by the higher frequency of the 'constitutional syndrome'; asthenia, anorexia, weight loss and other cancer-related symptoms (such as rectal bleeding) are prevalent in primary care, and their positive predictive value for cancer is low at that level (MacArthur and Smith, 1984; Joint Council for Clinical Oncology, 1993; Curless et al, 1994; Swenson et al, 1995).

At hospital admission, tumour stage was more advanced in the emergency group (Holliday and Hardcastle, 1979; Nilsson et al, 1982; Anderson et al, 1992; Hargarten et al, 1992; Scott et al, 1995), even though the symptom to diagnosis interval (or duration

 © Cancer Research Campaign 1998

of symptoms) was shorter in this group; this apparently paradoxical observation, which has been made before (Holliday and Hardcastle, 1979; Nilsson et al, 1982; Ratcliffe et al, 1989; Porta et al, 1991; Wile et al, 1993; Maguire et al, 1994; Mountney et al, 1994; Scott et al, 1995; Porta et al, 1996*b*), may essentially have two complementary explanations: firstly, the biological aggressiveness of tumours could on average be higher among patients that resort to the emergency department (Feinstein, 1966; Gómez et al, 1996; Piccirillo and Feinstein, 1996) and, secondly, these patients may underestimate the duration of their symptoms (e.g. a lower rate of physician contact may be associated with a lower awareness of symptoms). The finding that the duration of symptoms has little prognostic influence in cancers of the digestive tract has also been published previously (Holliday and Hardcastle, 1979; Nilsson et al, 1982; Brown et al, 1983; Ratcliffe et al, 1989; Haugstvedt et al, 1991; Porta et al, 1991; Wile et al, 1993; Maguire et al, 1994).

As in other settings (Haugstvedt et al, 1991; Anderson et al, 1992; Hargarten et al, 1992; Wile et al, 1993; Mountney et al, 1994; Mulcahy et al, 1994), treatment with a curative or radical intention was more common in electively admitted patients, and this was a strong determinant of prognosis. Nonetheless, even among subjects who underwent surgery, emergency patients had a 50–70% excess risk. It could be hypothesized that emergency patients were treated less often by senior surgeons (Gulliford et al, 1991; Anderson et al, 1992); yet, surgery within the first 2 days of admission was only slightly more common in emergency patients, and it did not carry an increased risk. Besides, a statistically significant excess risk of almost 60% persisted after adjusting for stage and type of initial treatment (models B and C, Table 4). Thus, the survival difference is unlikely to be explained by emergency patients having received suboptimal surgery in the emergency situation.

Overall, the excess risk observed among emergency patients was consistent enough to warrant further and serious consideration. This need is also supported by the limited scope of the literature in this area. Although from a clinical perspective it is known that patients with digestive tract cancer presenting electively have a higher chance of survival than those who present as true emergencies, comprehensive analyses are lacking. For example, the average proportion of cancers among all referrals is not available (Mountney et al, 1994), and population-based studies on health care utilization have not integrated data spanning from symptom onset to diagnosis and outcome. Specifically, an in-depth assessment of possible reasons and mechanisms for the difference in survival would require information in two areas: firstly, on the pre-hospital help-seeking and medical care process (e.g. health care choices made and management received by oncology patients, referral patterns) and, secondly, on the precise reasons for presentation to and admission through the emergency department (e.g. type of referral, presenting complaint, case severity). But even if comorbidity and severity of the disease at presentation could partly 'explain' the outcome differential, the clinically and policy-relevant fact would remain that the emergency patients' condition when they gain access to the hospital is too severe and thwarts survival.

Information on the in-hospital diagnostic and treatment process (timing of key clinical actions, indication for surgery, type of surgical procedure, subsequent therapeutic decisions, clinical course) could also be of interest. Nonetheless, we believe that the intrinsic quality of in-hospital care provided to emergency patients with cancer is essentially identical to that given to elective patients.

The above considerations outline the limitations of the present study. However, by contrast to clinically oriented studies, we were able to assess the role of a wide variety of factors through three types of data sources: personal interviews with patients provided data on the health care process followed from symptom onset to diagnosis; hospital clinical records furnished information on factors such as the diagnoses suspected at admission, stage and type of treatment; and the hospital tumour registry was instrumental in achieving a high rate of follow-up. Cancer registries have a role in assessing the need for services and clinical outcomes (Mountney et al, 1994; Calman, 1995; Malats et al, 1995; Selby et al, 1996). In fact, the analyses reported here were partly prompted by data from our hospital registry of a high rate of admission via the emergency department (Casamitjana et al, 1996; Porta et al, 1997).

The use of emergency departments for non-urgent care contributes to overcrowding and may hamper the quality of care that severely ill patients receive (Artal et al, 1991; Steinbrock, 1996). Yet, the policy implications of costs of non-urgent care in emergency departments are debated. Non-urgent visits to emergency departments partly reflect a failure to provide accessible primary care, particularly to minorities and to those of lower socioeconomic status (Hargarten, 1992; Tyrance et al, 1996). They also reflect severe problems in the integration between primary and secondary care. It could be argued that emergency departments constitute a useful resource for lessening diagnostic and therapeutic delays in cancer. However, the present study suggests that emergency departments are unable to counterbalance the deficiencies in the other levels of the health system.

What role can the primary care team realistically fulfil within the network of cancer services, particularly with respect to early detection, referral and follow-up? The Report by the Expert Advisory Group on Cancer to the Chief Medical Officers of England and Wales (Calman, 1995) advocates the integration of primary care with cancer units and centres. Whereas the report asserts that 'arrangements should be in place for rapid referral of patients to, or liaison with, a cancer unit or centre', it also acknowledges the need 'to allow flexibility for emergency presentations of cancers in hospitals without cancer units'. On a similar tone, an assessment review of health care needs for colorectal cancer concluded that 'targets could be set to reduce the current level of emergency admissions by up to 20%' (Mountney et al, 1994). Research and discussion on these and related issues is of paramount importance at a time when significant changes are underway in the organization of cancer services and of general practice – in Spain, in the UK and throughout Europe. Unless substantial progress is achieved, emergency admission for cancer shall remain a matter of survival.

### ACKNOWLEDGEMENTS

The authors are grateful to Francesc Macià, Xavier Fabregat, Montserrat Casamitjana, Cruz Molina, Manuel Jariod, Carme Borrell and Josep Planas for scientific support. Warm thanks are also due to Ignasi Tusquets, Jordi Carbonell, Juan Gérvas, Vicente Ortún and Antoni Sitges for their critiques of earlier versions of this paper. The excellent technical assistance provided by David J MacFarlane, Puri Barbas and the Pfeiffer Memorial Library staff in Milan is gratefully acknowledged. This work was partly funded by the Fondo de Investigación Sanitaria (grant 92/0311). Additional funding was provided by the Generalitat de Catalunya (CIRIT/1995 SGR 434). EF's stay at the 'Mario Negri' Institute was supported by a grant from the Human Capital and Mobility Research Training Programme of the European Union (contract no. ERBCHBGCT 930359).

© Cancer Research Campaign 1998

### REFERENCES

Anderson JH, Hole D and McArdle CS (1992) Elective *versus* emergency surgery for patients with colorectal cancer. *Br J Surg* **79**: 706–709

Armitage P and Berry G (1987) *Statistical Methods in Medical Research*, 2nd edn, pp. 125–132, 205–209. Blackwell: Oxford

Artal A, Garrido P, Berrocal A, Barón JM, Espinosa E, de la Gándara I and Juárez S (1991) Estudio descriptivo de la asistencia a enfermos oncológicos en el Servicio de urgencias de un hospital general. *Rev Clin Esp* **188**: 345–348

Belloc J, Porta M, Malats N, Gallén M and Planas J (1994) El síntoma inicial atribuible al cáncer en los tumores del tubo digestivo. Un análisis de la concordancia entre el paciente y el médico. *Med Clin* **103**: 401–407

Brown MW, Bradley JA and Calman KC (1983) Malignant disease in the accident and emergency department. *Br J Clin Pract* **37**: 205–208

Calman K (Chairman) (1995) *A Policy Framework for Commissioning Cancer Services. A Report by the Expert Advisory Group on Cancer to the Chief Medical Officers of England and Wales*. pp. 7–9, 27–29. Department of Health: London

Casamitjana M, Collet I, Fabregat X, Gallén M, Macià F, Malats N and Porta M (1996) *Informe del Registre de Tumors de l'Hospital del Mar, 1994*. Institut Municipal d'Assistència Sanitària: Barcelona

Clayton D and Hills M (1993) *Statistical Models in Epidemiology*. pp. 237–248, 271–306. Oxford University Press: Oxford

Collett D (1994) *Modelling Survival Data in Medical Research*. pp. 15–51, 53–106, 192–198. Chapman & Hall: London

Cox DR (1972) Regression models and lifetables (with discussion). *J R Statist Soc [B]* **34**: 187–220

Cox DR and Oakes D (1984) *Analysis of Survival Data*. pp. 1–28, 91–139. Chapman & Hall: London

Curless R, French JM, Williams GV and James OFW (1994) Colorectal carcinoma: do elderly patients present differently? *Age Ageing* **23**: 102–107

EGRET (1991) *Epidemiological Graphics, Estimation, and Testing Package*. Revision 3. Statistics and Epidemiology Research Corporation: Seattle

Feinstein AR (1966) Symptoms as an index of biological behaviour and prognosis in human cancer. *Nature* **209**: 241–245

Fielding LP, Phillips RKS and Hittinger R (1989) Factors influencing mortality after curative resection for large bowel cancer in elderly patients. *Lancet* **1**: 595–597

Gómez G, Porta M, Griful E, Maguire A, Calle ML, Malats N, Fernandez E, Piñol JL and Gallén M (1996) Modelling breast cancer survival and the symptom-to-treatment interval. *J Epidemiol Biostat* **1**: 175–182

Gulliford MC, Petruckevitch A and Burney PGJ (1991) Survival with bladder cancer, evaluation of delay in treatment, type of surgeon, and modality of treatment. *Br Med J* **303**: 437–440

Hargarten SW, Roberts MJS and Anderson AJ (1992) Cancer presentation in the emergency department: a failure of primary care. *Am J Emerg Med* **10**: 290–293

Haugstvedt TK, Viste A, Eide GE, Soreide O and Members of the Norwegian Stomach Cancer Trial (1991) Patient and physician treatment delay in patients with stomach cancer in Norway: is it important? *Scand J Gastroenterol* **26**: 611–619

Heys SD, Sherif A, Bagley JS, Brittenden J, Smart C and Eremin O (1994) Prognostic factors and survival of patients aged less than 45 years with colorectal cancer. *Br J Surg* **81**: 685–688

Holliday HW and Hardcastle JD (1979) Delay in diagnosis and treatment of symptomatic colorectal cancer. *Lancet* **1**: 309–311

Joint Council for Clinical Oncology (1993) *Reducing Delays in Cancer Treatment. Some Targets*. Royal College of Physicians and Royal College of Radiologists: London

Kalbfleisch JD and Prentice RL (1980) *The Statistical Analysis of Failure Time Data*. John Wiley: New York

Kaplan EL and Meier P (1958) Nonparametric estimation from incomplete observations. *J Am Statist Assoc* **53**: 457–481

Kleinbaum DG, Kupper LL and Morgenstern H (1982) *Epidemiologic Research. Principles and Quantitative Methods*. pp. 320–376, 419–491. Lifetime Learning Publications: Belmont, CA

MacArthur C and Smith A (1984) Factors associated with speed of diagnosis, referral, and treatment in colorectal cancer. *J Epidemiol Community Health* **38**: 122–126

Maguire A, Porta M, Malats N, Gallén M, Piñol JL and Fernandez E for the ISDS II Project Investigators (1994) Cancer survival and the duration of symptoms. An analysis of possible forms of the risk function. *Eur J Cancer* **30A**: 785–792

Malats N, Belloc J, Gallén M and Porta M (1995) Disagreement between hospital medical records and a structured patient interview on the type and date of the first symptom in cancer of the digestive tract. *Rev Épidémiol Santé Publique* **43**: 533–540

Mikulin T and Hardcastle JD (1987) Gastric cancer. Delay in diagnosis and its causes. *Eur J Cancer Clin Oncol* **23**: 1683–1690

Ministerio de Sanidad y Consumo (1995) *Estudio Territorializado de la Evolución del Sector Salud a partir de 1980*. pp. 186–188. Ministerio de Sanidad y Consumo: Madrid

Molina MC, Porta M, Malats N, Jariod M, Piñol JL and Fernandez E (1994) Percepción del inicio y la evolución de la sintomatología en pacientes hospitalizados con cáncer del tubo digestivo. *Neoplasia* **11**: 119–125

Mountney L, Sanderson H and Harris J (1994) Colorectal cancer. In: *Health Care Needs Assessment*, Vol. 1, Stevens A and Raftery J. (eds), pp. 379–410. Radcliffe Medical Press: Oxford

Mulcahy HE, Patchett SE, Daly L and O'Donoghue DP (1994) Prognosis of elderly patients with large bowel cancer. *Br J Surg* **81**: 736–738

Nilsson E, Bolin S and Sjödahl R (1982) Carcinoma of the colon and rectum. Delay in diagnosis. *Acta Chir Scand* **148**: 617–622

Piccirillo JF and Feinstein AR (1996) Clinical symptoms and comorbidity: significance for the prognostic classification of cancer. *Cancer* **77**: 834–842

Pope C (1993) Waiting times for outpatient appointments. *Br Med J* **306**: 408–409

Porta M, Gallén M, Malats N and Planas J (1991) Influence of "diagnostic delay" upon cancer survival: an analysis of five tumour sites. *J Epidemiol Community Health* **45**: 225–230

Porta M, Malats N, Belloc J, Gallén M and Fernandez E (1996*a*) Do we believe what patients say about their neoplastic symptoms? An analysis of factors that influence the interviewer's judgement. *Eur J Epidemiol* **12**: 553–562

Porta M, Gallén M, Belloc J and Malats N (1996*b*) Predictors of the interval between onset of symptoms and first medical visit in patients with digestive tract cancer. *Int J Oncol* **8**: 941–949

Porta M, Malats N, Morell E, Gomez G, Gallén M, Macià F, Casamitjana M and Fabregat X (1998) Decreased survival of patients with lung cancer admitted to a teaching hospital through the emergency department in Barcelona. *J Epidemiol Community Health* (in press)

Ratcliffe R, Kiff RS, Kingston RD, Walsh SH and Jeacock J (1989) Early diagnosis in colorectal cancer. Still no benefit? *J R Coll Surg Edinb* **34**: 152–155

Scott NA, Jeacock J and Kingston RD (1995) Risk factors in patients presenting as an emergency with colorectal cancer. *Br J Surg* **82**: 321–323

Selby P, Gillis C and Haward R (1996) Benefits from specialised cancer care. *Lancet* **348**: 313–318

Siegel S and Castellan NJ Jr (1988) *Nonparametric Statistics for the Behavioral Sciences*, 2nd edn. pp. 102–167, 190–223. McGraw-Hill: New York

Steinbrock R (1996) The role of the emergency department. *N Engl J Med* **334**: 657–658

Swenson KK, Rose MA, Ritz L, Murray CL and Adlis SA (1995) Recognition and evaluation of oncology-related symptoms in the emergency department. *Ann Emerg Med* **26**: 12–17

Tyrance PH Jr, Himmelstein DU and Woolhandler S (1996) US emergency department costs: no emergency. *Am J Public Health* **86**: 1527–1531

Vineis P, Fornero G, Magnino A, Giacometti R and Ciccone G (1993) Diagnostic delay, clinical stage, and social class: a hospital based study. *J Epidemiol Community Health* **47**: 229–231

West R and Rosen M (1996) United Kingdom study of urgent and emergency admission to hospital. *Health Trends* **28**: 13–19

Wile AG, Hourani L and Schell MJ (1993) The contributions of patient factors, physician delay, and tumor biology to the outcome of gastric cancer. *Am Surg* **59**: 850–854

© Cancer Research Campaign 1998